# Linear MALDI-ToF simultaneous spectrum deconvolution and baseline removal

**DOI:** 10.1186/s12859-018-2116-3

**Published:** 2018-04-05

**Authors:** Vincent Picaud, Jean-Francois Giovannelli, Caroline Truntzer, Jean-Philippe Charrier, Audrey Giremus, Pierre Grangeat, Catherine Mercier

**Affiliations:** 1 CEA, LIST, Laboratoire d’Intégration de Systèmes et des Technologies, DIGITEO Bât 565 - Point Courrier 192, Gif-sur-Yvette, 91191 Cedex France; 2 University of Bordeaux, IMS, UMR 5218, Talence, 33400 France; 3 CLIPP, Pôle de Recherche Université de Bourgogne, Dijon, 21000 France; 4 Technology Research Department, Innovation Unit, Marcy l’Étoile, bioMérieux SA, Marcy l’Étoile, 69280 France; 5 Université Grenoble Alpes, Grenoble, 38000 France; 6 CEA, LETI, MINATEC Campus, DTBS, 17 Rue des Martyrs, Grenoble, 38054 France; 7 Service de Biostatistique, Hospices Civils de Lyon, Lyon, 69000 France; 8 Université Lyon 1, Villeurbanne, 69100 France; 9 CNRS UMR5558, Laboratoire de Biométrie et Biologie Évolutive, Équipe Biostatistique-Santé, Villeurbanne, 69100 France

**Keywords:** Mass spectrometry, Peak picking, Deconvolution, Baseline

## Abstract

**Background:**

Thanks to a reasonable cost and simple sample preparation procedure, linear MALDI-ToF spectrometry is a growing technology for clinical microbiology. With appropriate spectrum databases, this technology can be used for early identification of pathogens in body fluids. However, due to the low resolution of linear MALDI-ToF instruments, robust and accurate peak picking remains a challenging task. In this context we propose a new peak extraction algorithm from raw spectrum. With this method the spectrum baseline and spectrum peaks are processed jointly. The approach relies on an additive model constituted by a smooth baseline part plus a sparse peak list convolved with a known peak shape. The model is then fitted under a Gaussian noise model. The proposed method is well suited to process low resolution spectra with important baseline and unresolved peaks.

**Results:**

We developed a new peak deconvolution procedure. The paper describes the method derivation and discusses some of its interpretations. The algorithm is then described in a pseudo-code form where the required optimization procedure is detailed. For synthetic data the method is compared to a more conventional approach. The new method reduces artifacts caused by the usual two-steps procedure, baseline removal then peak extraction. Finally some results on real linear MALDI-ToF spectra are provided.

**Conclusions:**

We introduced a new method for peak picking, where peak deconvolution and baseline computation are performed jointly. On simulated data we showed that this global approach performs better than a classical one where baseline and peaks are processed sequentially. A dedicated experiment has been conducted on real spectra. In this study a collection of spectra of spiked proteins were acquired and then analyzed. Better performances of the proposed method, in term of accuracy and reproductibility, have been observed and validated by an extended statistical analysis.

## Background

Linear matrix-assisted laser desorption/ionization time-of-flight mass spectrometry (MALDI-ToF MS) has now revolutionized identification of bacteria, yeasts and molds in clinical microbiology [1]. The technology is simple, accurate, fast, and for large laboratories less expensive than conventional methods. Despite a lower resolution than other analyzers used in modern proteomics, linear ToF are preferred in microbiology because of a better sensitivity in the 2-20 kDa mass range, where proteins contain phylogenic information. Moreover, the lower cost of linear instruments favored a wider adoption by health institutions. In essence, identifications are performed in minutes by simply acquiring an experimental spectrum of the whole microorganism cells and comparing the resulting peak list with a database [2, 3]. In this context we propose a new method for peak extraction especially adapted to linear MALDI-ToF spectra. The usual approach for MALDI mass spectra processing generally consists of chaining several procedures. Most of the times we have a smoothing step, a baseline correction step and only then the final the peak extraction [4]. The main idea of our new method is to jointly perform these steps with the aim of reducing the potential unrecoverable artifacts introduced by a sequential processing. In the next sections we briefly present the three main steps of the usual approaches. We then describe our method and its detailed derivation.

### Smoothing

A popular [5] smoothing technique in the spectrometry community is the use of Savitzky-Golay linear filters [6, 7]. These moving average filters perform a least squares fit of a small set of consecutive data points to a polynomial. The value of this fitted polynomial at the window central point is the filter output. One can also compute a smoothed derivative by using the derivative of the fitted polynomial to compute the central point value. This smoothed derivative can also be used by peak picking algorithms [4]. This method is versatile and efficient. However, its main drawbacks are that we have to manually choose the polynomial degree and the window length. Some studies to automatically choose the former [8] or later [9] exist but to the best of our knowledge they are not used as often as the original approach.

A more recent approach to smooth spectra is the use the wavelet transform. The Undecimated Wavelet Transform (UDWT) [10, 11] is generally preferred to the Discrete Wavelet Transform (DWT) as it produces less artifacts after coefficient thresholding. The UDWT is equivalent to an averaged DWT computed for all integer shifts of the signal and is thus a redundant and shift invariant transform. In applications it has been reported to yield better qualitative denoising [12].

### Baseline correction

Baseline correction is a difficult problem that potentially also introduces artifacts [13]. There are at least two kinds of approaches for baseline correction. One category of methods is close to mathematical morphology. In these methods a lower envelope of the spectrum [14, 15] is computed. Methods of this category generally need a smoothed signal (see “Smoothing” section). The other category contains methods using an asymmetric loss function to fit spectrum baseline without being biased by peaks [16, 17]. Finally some other methods mix the two previous approaches [18].

### Peak picking

After baseline removal the next step is generally a peak picking procedure. Several approaches are possible. Perhaps the most intuitive approach is to compute a regularized second order derivative (using Savitzky-Golay for instance) of the spectrum and to extract local minima [4]. The use of second order derivative minima instead of the zero-crossing of the first order derivative allows, to some extend, to detect overlapping peaks [19].

A second kind of approach, especially useful in case of overlapping peaks, is peak deconvolution. Overlapping of complex peak patterns can be deconvolved if one uses specially tuned point spread function and judicious regularizations (positivity constraint and sparsity-inducing norms, like the *l*_1_ norm) [20–23]. Further generalizations can be obtained in case of blind-deconvolution [24]. However these kinds of approaches are much more computationally intensive and are not widely used in mass spectrometry.

Finally we can mention the Continuous Wavelet Transform (CWT) [25, 26] which can be efficiently computed using the Fast Fourier Transform. The idea is to follow wavelet modulus maximum. Theses ridges characterize the regularity of the signal [27] and can be used to detect peaks.

## Contributions

Computing the baseline correction and finding peaks are two strongly linked problems, it is thus natural to perform these two operations jointly. In this work we propose such an approach.

In the first part of the paper we describe a direct model with an additive noise where the spectrum is modelized by a smooth baseline plus a sparse peak list convolved by a given peak shape function. We describe how we chose our priors to enforce baseline smoothness and sparsity of the peak list. We then assume a Gaussian distribution for the noise. This allows us to use Euclidean distance to quantify the error between our model and the measured spectrum.

Next we show how the unknown baseline can be eliminated from the model. This manipulation leads to a modified problem very close to the classical deconvolution one. We underline this similarity and rigorously describe the two limiting cases, zero or infinite penalization for the baseline smoothness. As a by product we can interpret that our new deconvolution method is equivalent in some way to deconvolve a regularized second order derivative of the initial spectrum.

We then carefully examine the behavior of the computed baseline at the spectrum boundaries. We observed that when the smoothness penalty is too strong, the computed baseline can become overly flat. To avoid this effect a correction allowing to define baseline values at boundaries is proposed. With this modification the behavior of the baseline at the boundaries is no more affected by strong baseline smoothness penalty.

An effective optimization method to compute the solution of the deconvolution problem is exposed. This optimization algorithm is used twice in our two-passes deconvolution procedure. In the first pass a sparsity prior is used and a first optimization problem is solved to find peak centers. In the second pass the sparsity prior is replaced by the previously found peak positions. This second optimization problem is solved to compute peak height values.

Finally the new method is compared to one instance of the smoothing/baseline correction/deconvolution classical approach. The advantage of the joint baseline computation and peak deconvolution is demonstrated on synthetic data. An example on “real” data is shown and a reference to a more detailed comparison between our method and classical ones is given.

## Method

### Problem definition

The proposed developments are founded on a natural model for the observed spectrum **y** as a spiky signal **x**_*p*_ convolved with a peak shape *p* superimposed onto a smooth baseline **x**_*b*_

1 $$ \mathbf{y} = \mathbf{x}_{b} + \mathbf{x}_{p} \ast p + \mathbf{e}  $$

where **e** includes measurement and model errors. It is a common linear model with additive uncertainties. These quantities are represented by vectors of size *n*, where *n* is the number of *m*/*z* channels of the original spectrum **y**.

The problem at stake is to recover both the signal of interest **x**_*p*_ and the baseline **x**_*b*_. It is a difficult task at least for three reasons.

- It is underdetermined since the number of unknowns is twice the number of data.
- The convolution reduces the resolution due to peak enlargement and possible overlap.
- Measurement noise and possible model inadequacy induce additional uncertainties.

As a consequence, information must be accounted for regarding the expected signals **x**_*p*_ and **x**_*b*_. In the following developments, **x**_*b*_ is expected to be smooth while **x**_*p*_ is expected to be spiky and positive. This knowledge will be included in the next sections.

#### **x**_*b*_ smoothness

A simple way to account for smoothness of **x**_*b*_ is to penalize its fluctuations through

2 $$  \mathcal{P}_{b}\left(\mathbf{x}_{b}\right) = \frac{\mu}{2} \sum_{k=1}^{n-1} \left(\mathbf{x}_{b}\left[k+1\right] - \mathbf{x}_{b}\left[k\right]\right)^{2} = \frac{\mu}{2} \|\mathbf{Dx}_{b}\|^{2}_{2}  $$

where **D** is a finite differences matrix of size (*n*−1)×*n* (given in Appendix “Smoothness and convolution matrix”) and *μ*>0.

#### **x**_*p*_ sparsity and positivity

In order to favor sparsity for **x**_*p*_ (spiky property) an elastic-net penalty is introduced

3 $$ \mathcal{P}_{p}(\mathbf{x}_{p}) = \lambda_{1} \big\|\mathbf{x}_{p}\big\|_{1} + \frac{\lambda_{2}}{2} \big\|\mathbf{x}_{p}\big\|_{2}^{2}  $$

The degree of sparsity is controlled by *λ*_1_. The coefficient *λ*_2_ is generally set to zero or to a very small value. The reason why we have introduced this extra regularization is that a small positive value can sometimes improve convergence speed of the algorithm. In practice this only happens for spectra of several thousand of *m*/*z* channels and always has a limited impact on the obtained solution.

In addition, the solution also requires positivity of **x**_*p*_, i.e. positivity for each component **x**_*p*_[ *k*]. The *l*_1_ norm then simplifies

$$\|\mathbf{x}_{p}\|_{1} = \sum_{k=1}^{n} \mathbf{x}_{p}\left[k\right] = \mathbb{1}_{n}^{t} \, \mathbf{x}_{p} $$

where $\mathbb {1}_{n}$ is the *n* dimensional column vector with each entry to 1. This substitution turns the convex, but nonsmooth, penalty $\mathcal {P}_{p}(\mathbf {x}_{p})$ into a smooth quadratic one. This idea has already been used in [23, 28].

#### Data-fidelity term

A common and natural way to quantify data - model discrepancy founded on Eq. 1 relies on a squared Euclidean norm:

4 $$ \mathcal{J}_{\text{obs}}\left(\mathbf{x}_{b},\mathbf{x}_{p}\right) = \frac{1}{2} \big\|\mathbf{y} - \left(\mathbf{x}_{b} + \mathbf{Lx}_{p}\right) \big\|^{2}_{2}  $$

where the *n*×*n* band matrix **L** represents the convolution with the peak shape *p*.

#### Complete objective

Using Eqs. 2, 3 and 4, we get a first expression of the complete objective:

5 $$ \mathcal{J}\left(\mathbf{x}_{b},\mathbf{x}_{p}\right) = \mathcal{J}_{\text{obs}}\left(\mathbf{x}_{b},\mathbf{x}_{p}\right) + \mathcal{P}_{b}\left(\mathbf{x}_{b}\right) + \mathcal{P}_{p}\left(\mathbf{x}_{p}\right)  $$

that is to say:

6 $$\begin{array}{*{20}l} \mathcal{J}\left(\mathbf{x}_{b},\mathbf{x}_{p}\right) &= \frac{1}{2} \|\mathbf{y} - \left(\mathbf{x}_{b} + \mathbf{Lx}_{p}\right) \|^{2}_{2} \\ & \quad+ \frac{\mu}{2} \|\mathbf{D}\mathbf{x}_{b}\|^{2}_{2}+ \lambda_{1} \|\mathbf{x}_{p}\|_{1} + \frac{\lambda_{2}}{2} \|\mathbf{x}_{p}\|_{2}^{2}  \end{array} $$

The minimization of this objective function

7 $$ \left(\widehat{\mathbf{x}}_{p},\widehat{\mathbf{x}}_{b}\right) = ~\underset{\mathbf{x}_{p}\ge0\,,\,\mathbf{x}_{b}}{\mathrm{arg\,min}}~\mathcal{J}\left(\mathbf{x}_{b},\mathbf{x}_{p}\right)  $$

gives the desired solution $\left (\widehat {\mathbf {x}}_{p},\widehat {\mathbf {x}}_{b}\right)$.

#### Elimination of **x**_*b*_

To find the solution of problem Eq. 7 we begin by solving it for the **x**_*b*_ vector. This is an unconstrained quadratic problem and an analytical solution can be found. We shall first write down the gradient of $\mathcal {J}$ with respect to **x**_*b*_:

8 $$ \nabla_{\mathbf{x}_{b}}\mathcal{J} = \left(\mathbf{I}_{b} + \mu \mathbf{D}^{t}\mathbf{D}\right) \mathbf{x}_{b} - \left(\mathbf{y} - \mathbf{L}\mathbf{x}_{p}\right)  $$

Solving $\nabla _{\mathbf {x}_{b}} \mathcal {J} =0$ yields the minimizer:

9 $$\begin{array}{*{20}l} \widehat{\mathbf{x}}_{b} &=\,\,\left(\mathbf{I}_{b}+\mu \mathbf{D}^{t}\mathbf{D}\right)^{-1}\left(\mathbf{y}-\mathbf{L}\mathbf{x}_{p}\right)  \\ &=\,\, \mathbf{B}_{\mu}^{-1}\left(\mathbf{y}-\mathbf{L}\mathbf{x}_{p}\right) \end{array} $$

where $\mathbf {B}_{\mu }=\mathbf {I}_{b}+\mu \mathbf {D}^{t}\mathbf {D}$. This operation is always possible since **B**_*μ*_ is invertible (sum of the identity matrix and a semi-positive matrix).

It is then possible to substitute **x**_*b*_ for $\widehat {\mathbf {x}}_{b}$ in objective Eq. 5 to obtain a reduced objective. After some algebra (proof is given in Appendix “Derivation of reduced objective”), we have

$$\widetilde{\mathcal{J}}(\mathbf{x}_{p}) = \mathcal{J}\left(\widehat{\mathbf{x}}_{b},\mathbf{x}_{p}\right) + \mathbf{C} $$

where **C** is a constant, and $\widetilde {\mathcal {J}}(\mathbf {x}_{p})$ is defined by:

10 $$\begin{array}{@{}rcl@{}} \widetilde{\mathcal{J}}(\mathbf{x}_{p}) & = & \frac{1}{2} \mathbf{x}_{p}^{t} \, \left(\mathbf{L}^{t}\mathbf{A}_{\mu} \mathbf{L} + \lambda_{2} \mathbf{I}_{b} \right)\, \mathbf{x}_{p}  \\ & & - \mathbf{x}_{p}^{t} \, \left(\mathbf{L}^{t}\mathbf{A}_{\mu} \mathbf{y} - \lambda_{1} \mathbb{1}_{n}\right)  \end{array} $$

The matrix **A**_*μ*_ is defined by:

11 $$ \mathbf{A}_{\mu} = \mathbf{I}_{b}-\mathbf{B}_{\mu}^{-1} = \mathbf{I}_{b}-\left(\mathbf{I}_{b}+\mu \mathbf{D}^{t}\mathbf{D}\right)^{-1}  $$

and its interpretation is discussed in detail in “Analysis of the **A**_*μ*_ matrix” section).

This quadratic form $\widetilde {\mathcal {J}}(\mathbf {x}_{p})$ is our objective function and the solution $\widehat {\mathbf {x}}_{p}$ is its minimizer subject to positivity:

12 $$ \widehat{\mathbf{x}}_{p} = \underset{\mathbf{x}_{p}\ge 0}{\mathrm{arg\,min}} \widetilde{\mathcal{J}}(\mathbf{x}_{p})  $$

Once this constrained quadratic problem is solved, we can retrieve $\widehat {\mathbf {x}}_{b}$ using Eq. 9:

$$\widehat{\mathbf{x}}_{b} = \mathbf{B}_{\mu}^{-1} \left(\mathbf{y}-\mathbf{L} \, \widehat{\mathbf{x}}_{p}\right) $$

then the initial problem Eq. 7 is solved.

The gradient of $\widetilde {\mathcal {J}}(\mathbf {x}_{p})$ is easily deduced form Eq. 10 and reads:

13 $$  \nabla_{\mathbf{x}_{p}}\widetilde{\mathcal{J}} = \left(\mathbf{L}^{t}\mathbf{A}_{\mu} \mathbf{L} + \lambda_{2} \mathbf{I}_{p}\right) \mathbf{x}_{p} - \left(\mathbf{L}^{t}\mathbf{A}_{\mu} \mathbf{y} - \lambda_{1} \mathbb{1}_{p}\right)  $$

### Interpretation of the reduced criterion

We can make a pause here to interpret Eq. 12. If there was no baseline a natural way to deconvolve the spectrum would be to solve:

$$\underset{\mathbf{x}_{p}\ge0}{\mathrm{arg\,min}} \frac{1}{2}\big\|\mathbf{y} - \mathbf{L}\mathbf{x}_{p}\big\|^{2}_{2} + \lambda \big\|\mathbf{x}_{p}\big\|_{1} $$

If we use the positivity constraint and expand this objective function we get the following equivalent problem:

14 $$ \underset{\mathbf{x}_{p}\ge0}{\mathrm{arg\,min}} \frac{1}{2}\mathbf{x}_{p}^{t} \mathbf{L}^{t}\mathbf{L}\mathbf{x}_{p} + \mathbf{x}_{p}^{t}\left(\lambda_{1} \mathbb{1}_{p} - \mathbf{L}^{t}\mathbf{y}\right)  $$

Now if we look Eq. 10 and set *λ*_2_=0 we get:

15 $$ \underset{\mathbf{x}_{p}\ge0}{\mathrm{arg\,min}} \frac{1}{2}\mathbf{x}_{p}^{t} \mathbf{L}^{t}\mathbf{A}_{\mu} \mathbf{L}\mathbf{x}_{p}+\mathbf{x}_{p}^{t}(\lambda_{1} \mathbb{1}_{p} - \mathbf{L}^{t}\mathbf{A}_{\mu} \mathbf{y})  $$

The two equations are very similar apart from the fact that the operator **A**_*μ*_ is applied to the reconstructed peaks **Lx**_*p*_ and to the raw spectrum **y**. It could be interpreted as the precision (inverse of the covariance) matrix in a correlated noise framework. Instead of this classical approach and to better understand its role in our deconvolution context we study the evolution of **A**_*μ*_ in the two limiting cases $\mu \rightarrow 0$ and $\mu \rightarrow \infty $.

### Analysis of the **A**_*μ*_ matrix

**Fig. 1:**
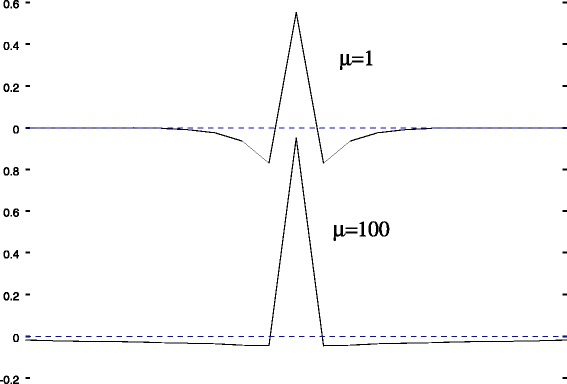
**A**_*μ*_ operator. $\mathbf {A}_{\mu }\left (:,\lfloor {\frac {n}{2}}\rfloor \right)$ column plot for *μ*=1 and *μ*=100. **A**_*μ*_ operator acts like a regularized second order derivation. The regularization strength increases as *μ* increases (*μ* is the background smoothness parameter)

Figure 1 shows the column $j=\lfloor {\frac {n}{2}}\rfloor $ of **A**_*μ*_ for two different values of *μ*.

For small *μ*, the **A**_*μ*_ operator acts like a regularized second order derivation. For a sufficiently small *μ* (such that the spectral radius satisfies *ρ*(*μ***D**^*t*^**D**)<1) a Taylor expansion gives:

$$\begin{array}{*{20}l} \mathbf{A}_{\mu} &\, =\,\, \mathbf{I}_{b}-\left(\mathbf{I}_{b}+\mu \mathbf{D}^{t}\mathbf{D}\right)^{-1}  \\ &\, =\,\, \mathbf{I}_{b}-\left(\mathbf{I}_{b}-\mu \mathbf{D}^{t}\mathbf{D}+o(\mu)\right)  \\ &\, =\,\, \mu \mathbf{D}^{t}\mathbf{D} +o(\mu) \end{array} $$

Hence compared to Eq. 14, one can interpret Eq. 15 as an usual deconvolution applied on the second order derivative **A**_*μ*_**y** of the initial spectrum **y**. The used point spread function is also the second order derivative **A**_*μ*_**L** of the initial peak shape *p* introduced in Eq. 1. The overall effect of the **A**_*μ*_ operator is to cancel the slow varying component of the signal. In another terms, the baseline is removed thanks to a derivation.

The regularization strength increases as the baseline parameter *μ* increases. At the limit $\mu \rightarrow \infty $ (proof given in Appendix “[Sec Sec36]”) we get:

$$\lim\limits_{\mu\rightarrow\infty} \left[\mathbf{A}_{\mu}\right]_{i,j} = \delta_{i,j}-\frac{1}{n} $$

When applied to any vector **y** we get

$$\lim\limits_{\mu\rightarrow\infty} \mathbf{A}_{\mu}\mathbf{y} = \mathbf{y} - \bar{\mathbf{y}}\mathbb{1}_{n} $$

Thus the action of **A**_*μ*_ centers the signal **y** by subtracting the constant $\bar {\mathbf {y}}\mathbb {1}_{n}$ vector, where the scalar $\bar {\mathbf {y}}$ is the mean value of the **y** vector’s components. The same centering holds for the peak shape function *p*. It follows that in this limiting case the solved problem is the usual deconvolution procedure applied on the centered spectrum. The computed baseline is simply a constant equal to the spectrum mean.

### Debiasing

The *l*_1_ penalty acts as a soft threshold to select peaks ([29], Section 10). This leads to a bias in peak intensity estimation. These intensities are artificially reduced when the *l*_1_ penalty increases. We use the ideas introduced in [28] to get corrected peak intensities. This yields a resolution procedure involving two stages. The first stage selects the peaks, the second one corrects their intensities.

#### First stage: peak support selection

Given *μ*, *λ*_1_ and *λ*_2_, we solve the minimization problem Eq. 12. The obtained solution $\widehat {\mathbf {x}}_{p}$ is a sparse vector containing peak intensities. The intensities are biased if the *λ* hyper-parameters are not null. However we can use $\widehat {\mathbf {x}}_{p}$ to define the peak support *Ω*. In the present work the peak support is simply defined by keeping the local maxima of $\widehat {\mathbf {x}}_{p}$.

16 $$\begin{array}{@{}rcl@{}} \Omega & = & \left\{ i, \left(\left(\widehat{\mathbf{x}}_{p}[\!i]>\widehat{\mathbf{x}}_{p}[\!i-1]\right)\wedge\left(\widehat{\mathbf{x}}_{p}[\!i]\ge\widehat{\mathbf{x}}_{p}[\!i+1]\right)\right)\vee \right. \\ & & \left.\left(\left(\widehat{\mathbf{x}}_{p}[\!i]\ge\widehat{\mathbf{x}}_{p}[\!i-1]\right)\wedge\left(\widehat{\mathbf{x}}_{p}[\!i]>\widehat{\mathbf{x}}_{p}[\!i+1]\right)\right) \right\} \end{array} $$

The peak intensities are going to be corrected in the second stage, but their final positions are defined by the condition Eq. 16. More complex procedures can be used to find the peak support. One such example is the procedure presented in [22], *Post-processing and thresholding*. These more refined methods can be introduced in a straightforward way in our approach by using them instead of the basic condition Eq. 16.

#### Second stage: peak intensity correction

In the second step, as we have peak support *Ω*, we do not need the *l*_1_ regularization anymore. Hence we simply ignore it and solve the simplified optimization problem:

17 $$\begin{array}{*{20}l} \mathbf{x}_{p} &\,=\,\, {\mathrm{arg\,min}} \frac{1}{2} \mathbf{x}_{p}^{t} \mathbf{L}^{t}\mathbf{A}_{\mu} \mathbf{L} \mathbf{x}_{p} - \mathbf{x}_{p}^{t} \mathbf{L}^{t}\mathbf{A}_{\mu} \mathbf{y}  \\ & \mathtt{s.t.} \quad \left\{\begin{array}{rcl} \mathbf{x}_{p}[\!k]=0 & \text{\texttt{if}} & k\notin\Omega \\ \mathbf{x}_{p}[\!k]\ge0 & \text{\texttt{if}} & k\in\Omega \end{array} \right. \end{array} $$

Despite its more complex appearance, this problem is no more complicated than Eq. 12. Solving this problem will correct peak intensities by removing the bias induced by the previously used *l*_1_ penalty.

### Boundary conditions

**Fig. 2:**
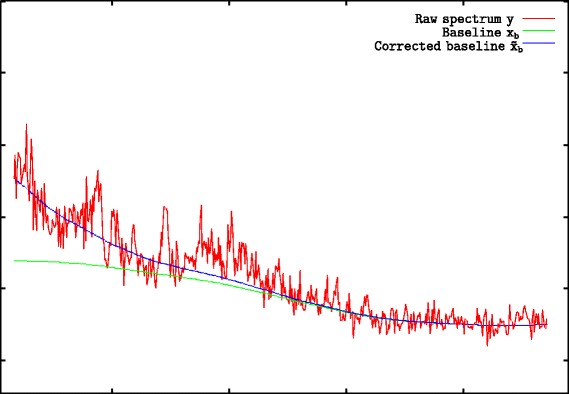
The boundary problem and its correction The green curve is the baseline **x**_*b*_ as computed by Eq. 2, the blue curve is the baseline $\tilde {x}_{b}$ after boundary condition correction

There is a possible improvement of the method concerning boundary conditions. As Eq. 2 suggests, a strong *μ* penalty forces the baseline to be constant. In Fig. 2 we see that this phenomenon is especially present at the domain boundaries.

We solved this problem by imposing baseline values at boundaries. This corrected solution is also shown in Fig. 2 and we can see that the corrected solution does not suffer from boundary effect anymore. Appendix “Boundary correction”, page 11, provides all the details on how to modify Eqs. 9 and 13 to introduce some constraints on the baseline values **x**_*b*_. These modified equations will constitute our final model formulation.

### Final model formulation

After boundary correction, the modified Eq. 9 is

18 $$ \mathbf{x}_{b} = \widetilde{\mathbf{B}}_{\mu}^{-1}\left(\widetilde{\mathbf{y}}-\mathbf{L}\mathbf{x}_{p}\right)  $$

and the modified Eq. 10 is

19 $$\begin{array}{*{20}l}  \widetilde{\mathcal{J}}_{2}(\mathbf{x}_{p}) =\quad & \frac{1}{2} \mathbf{x}_{p}^{t}\left(\lambda_{2} \mathbf{I}_{p}+ \mathbf{L}^{t}\widetilde{\mathbf{A}}_{\mu} \mathbf{L}\right) \mathbf{x}_{p}+  \\ & \, \mathbf{x}_{p}^{t}\left(\lambda_{1} \mathbf{\mathbb{1}}_{p} - \mathbf{L}^{t}\widetilde{\mathbf{A}}_{\mu}\widetilde{\mathbf{y}} - \mathbf{L}^{t} (\mathbf{y}-\widetilde{\mathbf{y}})\right) \end{array} $$

The solution $\widehat {\mathbf {x}}_{p}$ is the unique minimizer of $\widetilde {\mathcal {J}}_{2}$ subject to positivity constraint:

20 $$ \widehat{\mathbf{x}}_{p} = \underset{\mathbf{x}_{p}\ge 0}{\mathrm{arg\,min}} \widetilde{\mathcal{J}}_{2}(\mathbf{x}_{p})  $$

The gradient is obtained by a straightforward computation:

21 $$  \nabla_{\mathbf{x}_{p}} \widetilde{\mathcal{J}}_{2} = \left(\lambda_{2} \mathbf{I}_{p}+ \mathbf{L}^{t}\widetilde{\mathbf{A}}_{\mu} \mathbf{L}\right) \mathbf{x}_{p} + \lambda_{1} \mathbf{\mathbb{1}}_{p} - \mathbf{L}^{t}\widetilde{\mathbf{A}}_{\mu}\widetilde{\mathbf{y}} - \mathbf{L}^{t} (\mathbf{y}-\widetilde{\mathbf{y}})  $$

As before, $\widehat {\mathbf {x}}_{b}$ is computed from $\widehat {\mathbf {x}}_{p}$ using Eq. 18. The explicit forms of $\widetilde {\mathbf {B}}_{\mu }$, $\widetilde {\mathbf {y}}$ and $\widetilde {\mathbf {A}}_{\mu }$ are given in Appendix “Boundary correction”, respectively by Eqs. 31, 32 and 33.

### Algorithm summary

For ease of reading Algorithm 1 recapitulates the main steps of the proposed method in its final formulation. The optimization procedure used to solve efficiently the two minimization problems will be described in detail in the next section.



## Effective minimization

### Quadratic programming with bound constraints

To solve the optimization problems Eq. 12 or Eq. 20 and their associated debiasing step Eq. 17 we use the projected Barzilai-Borwein method described in [30]. The Barzilai-Borwein method [31] dramatically improves the classical steepest descent with Cauchy step.

For a convex quadratic form

$$\begin{array}{*{20}l} \mathbf{x}_{p} & = \underset{\mathbf{x}}{\mathrm{arg\,min}} \mathcal{J}(\mathbf{x}) \\ & = \underset{\mathbf{x}}{\mathrm{arg\,min}} \frac{1}{2} \mathbf{x}^{t} \mathbf{Q} \mathbf{x} + \mathbf{q}^{t}\mathbf{x} \end{array} $$

the steepest descent direction is defined by

$$\mathbf{x}^{(k+1)}=\mathbf{x}^{(k)}-\alpha \nabla_{\mathbf{x}^{(k)}} \mathcal{J} $$

with

$$\nabla_{\mathbf{x}^{(k)}}\mathcal{J}=\mathbf{Q} \mathbf{x}^{(k)} + \mathbf{q} $$

The associated Cauchy step is defined by

$$\alpha_{\text{Cauchy}} = \arg\min\limits_{\alpha} \mathcal{J}\left(\mathbf{x}^{(k)}-\alpha \nabla_{\mathbf{x}^{(k)}} \mathcal{J}\right) $$

A straightforward calculation leads to:

$$\alpha_{\text{Cauchy}}=\frac{\| \nabla_{\mathbf{x}^{(k)}} \mathcal{J} \|^{2}_{2}}{\nabla_{\mathbf{x}^{(k)}} \mathcal{J}^{t}\, \mathbf{Q}\, \nabla_{\mathbf{x}^{(k)}} \mathcal{J}} $$

The Barzilai-Borwein method uses the same steepest descent direction but replaces the Cauchy step by one (or an alternating sequence) of the two so-called Barzilai-Borwein steps. These step lengths are defined by [30]:

22 $$ \alpha_{BB1}= \frac{\big\|\mathbf{x}^{(k)}-\mathbf{x}^{(k-1)}\big\|^{2}_{2}}{\left(\mathbf{x}^{(k)}-\mathbf{x}^{(k-1)}\right)^{t}\,\left(\nabla_{\mathbf{x}^{(k)}} \mathcal{J}-\nabla_{\mathbf{x}^{(k-1)}} \mathcal{J}\right)}  $$

23 $$  \alpha_{BB2}= \frac{\left(\mathbf{x}^{(k)}-\mathbf{x}^{(k-1)}\right)^{t}\,\left(\nabla_{\mathbf{x}^{(k)}} \mathcal{J}-\nabla_{\mathbf{x}^{(k-1)}} \mathcal{J}\right) }{\big\|\nabla_{\mathbf{x}^{(k)}} \mathcal{J}-\nabla_{\mathbf{x}^{(k-1)}} \mathcal{J}\big\|^{2}_{2}}  $$

Despite its simple update formula the Barzilai-Borwein method works surprisingly well [32, 33]. This method has no line search and exhibits a non-monotonic convergence. It has been shown to be globally convergent for the strictly convex quadratic case [34]. A direct extension to the constrained case is obtained by replacing the iterate **x**^(*k*+1)^ by its projection on the feasible domain:

24 $$ \mathbf{x}^{(k+1)}=P\left(\mathbf{x}^{(k)}-\alpha \nabla_{\mathbf{x}^{(k)}} \mathcal{J}\right)  $$

For bound constraints **x**∈ [ **l**,**u**] the projection operator *P* is simply defined (in a component wise fashion) by

$$P(\mathbf{x})=\min\left(\mathbf{u},\max(\mathbf{l},\mathbf{x})\right) $$

Without any line search [30] gives a counter-example where this method is not convergent. However in practice the method is generally successful, especially for badly conditioned problems where it can outperform the constrained conjugate gradients algorithm [35].

For its simplicity and good behavior in practice we have chosen to use this method. Our implementation is given in pseudo-code in Algorithm 2.



Variants of this method with non-monotone line search [36, 37] have been tested. Theoretically this allows to prove global convergence, but in practice we have observed a performance degradation compared to the simpler method presented in [30].

### Stopping criterion

Algorithm 2 stopping criterion needs to be defined. We use a rigorous one based on Karush-Kuhn-Tucker (KKT) conditions [38]. For a smooth convex optimization problem

$$\mathbf{x}_{p} = \underset{\mathbf{x}\in[\mathbf{l},\mathbf{u}]}{\mathrm{arg\,min}} \mathcal{F}(\mathbf{x}) $$

necessary and sufficient conditions are:

1. Stationarity $\nabla _{\mathbf {x}_{p}} \mathcal {F}+\mathbf {\lambda }^{\mathbf {u}}-\mathbf {\lambda }^{\mathbf {l}}=\mathbf {0}$
2. Primal feasibility $\mathbf {x}_{p} \in \left [\mathbf {l},\mathbf {u}\right ]$
3. Dual feasibility
  Lower bounds **λ**^**l**^≥**0**
  Upper bounds **λ**^**u**^≥**0**
4. Complementary slackness
  Lower bounds $\forall i,\ \mathbf {\lambda }^{\mathbf {l}}\left [i\right ](\mathbf {l}\left [i\right ]-\mathbf {x}_{p}\left [i\right ])=0$
  Upper bounds $\forall i,\ \mathbf {\lambda }^{\mathbf {u}}\left [i\right ](\mathbf {x}_{p}\left [i\right ]-\mathbf {u}\left [i\right ])=0$

Our stopping criterion reflects KKT conditions violation. Enumerating all possible cases this criterion is computed as follow

$$\text{stopping criterion} = \sum_{i} |\mathbf{s}\left[i\right]|$$

with **s**[ *i*] defined by:

25 $$ {}{\mathbf{s}\left[i\right]= \left\{ \begin{array}{ccc} +\infty & \text{if} & \mathbf{x}\left[i\right]\notin \left[\mathbf{l}\left[i\right],\mathbf{u}\left[i\right]\right] \\ \min\left(0,\nabla_{\mathbf{x}_{p}} \mathcal{F}\left[i\right]\right) & \text{if} & \left(\mathbf{x}\left[i\right] = \mathbf{l}\left[i\right]\right) \wedge \left(\mathbf{l}\left[i\right]<\mathbf{u}\left[i\right]\right) \\ \max\left(0,\nabla_{\mathbf{x}_{p}} \mathcal{F}\left[i\right]\right) & \text{if} & \left(\mathbf{x}\left[i\right] = \mathbf{u}\left[i\right]\right) \wedge \left(\mathbf{l}\left[i\right]<\mathbf{u}\left[i\right]\right) \\ \nabla_{\mathbf{x}_{p}} \mathcal{F}\left[i\right] & \text{if} & \mathbf{x}\left[i\right]\in ] \mathbf{l}\left[i\right],\mathbf{u}\left[i\right] [ \\ 0 & \text{if} & \mathbf{l}\left[i\right]=\mathbf{x}\left[i\right]=\mathbf{u}\left[i\right] \\ \end{array} \right.}  $$

### Illustration of the method

**Fig. 3:**
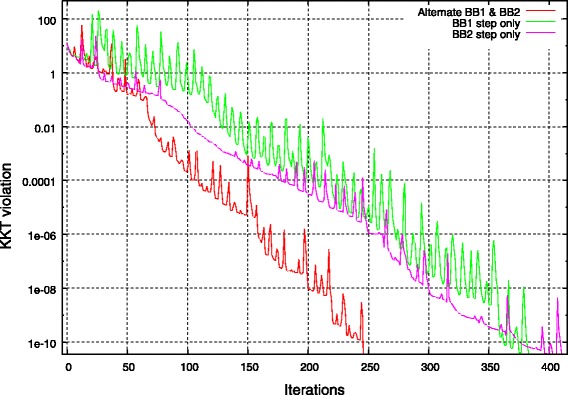
Typical convergence behavior of the **PBB** algorithm given in Algorithm 2. Alternating between BB1 and BB2 steps leads to faster convergence

We give in Fig. 3 typical convergence behavior for the proposed method applied to problem Eq. 12 or Eq. 17.

We observe the non-monotonic convergence behavior of the Barzilai-Borwein method. As reported by [30], we observe that it is more effective to alternate between BB1 Eq. 22 and BB2 Eq. 23 steps than using only one type of step update.

## Results and discussion

### Synthetic data

The presented algorithm has been tested on synthetic data. The synthetic spectra consist of **y** vectors of *n*=500 components. Each **y** is generated by summing contributions from a synthetic baseline **x**_*b*_, a synthetic peak list **x**_*p*_ convolved by a Gaussian peak shape *p* and a Gaussian noise **ε**:

$$\mathbf{y}=\mathbf{x}_{b} + \mathbf{x}_{p}\ast p + \mathbf{\epsilon} $$

The detailed description of these three contributions follows.

- **Synthetic baseline**
  The analytic expression of the baseline is
  $$\mathbf{x}_{b}\left[i\right] = C(s)+s\exp{\left(-3\frac{i}{n}\right)}-2\frac{i}{n} $$
  where *s* can take one of the following values *s*=−1,0,or 1.
  For *s*=0 the baseline is a straight line, for *s*=−1 the baseline is concave, for *s*=+1 it is convex.
  The constant *C*(*s*) insure a positive spectrum. Its value is *C*(*s*)=5 for *s*=1 and *C*(*s*)=2 otherwise.
- **Synthetic peak list**
  The analytic expression of the peak list contribution is:
  $$\left(\mathbf{x}_{p}\ast p\right)[\!i]=\sum\limits_{k=1}^{n_{p}}\alpha_{k} p\left(\mu_{k},\sigma_{p},i\right) $$
  where we have taken *n*_*p*_=10 and
  $$p\left(\mu_{k},\sigma_{p},i\right)= \exp{\left(-\frac{1}{2}\left(\frac{i-\mu_{k}}{\sigma_{p}}\right)^{2}\right)} $$
  These *n*_*p*_ Gaussian peaks are defined by a constant shape factor *σ*_*p*_=10. The individual heights *α*_*k*_ and centers *μ*_*k*_ are tabulated in Table 1.

**Table 1:** True peak centers *μ*_*k*_ and intensities *α*_*k*_. All peaks have a common shape factor *σ*_*p*_=10

*k*	*μ*_*k*_	*α*_*k*_
1	50	1
2	90	0.5
3	170	0.5
4	200	3
5	230	2
6	260	1
7	350	0.5
8	370	3
9	390	2
10	410	1
- **Simulated zero mean Gaussian noise**
  The noise **ε** follows a Normal law of zero mean and *σ*_*noise*_ standard deviation:
  $$\mathbf{\epsilon}[\!i] \sim \mathcal{N}\left(0,\sigma_{noise}\right) $$

### Algorithm implementation

We provide a reference implementation^1^ that can be used to reproduce the results of the following sections. This implementation is coded in C++ and runs under Linux. The main page of the project details all the steps to reproduce the results of the “Joint baseline computation-peak deconvolution”, “Comparison with the usual sequential approach” and “Real data” sections. Typical run-times are one second for the synthetic data and three seconds for the examples using real spectra data. To keep this implementation as simple as possible a basic projected gradient descent is used instead of the more effective Algorithm 2. Also note that this implementation requires CSV input files. A more versatile version of our algorithm, using Algorithm 2, is used in [39]. However, this implementation is not yet publicly available.

### Joint baseline computation-peak deconvolution

**Fig. 4:**
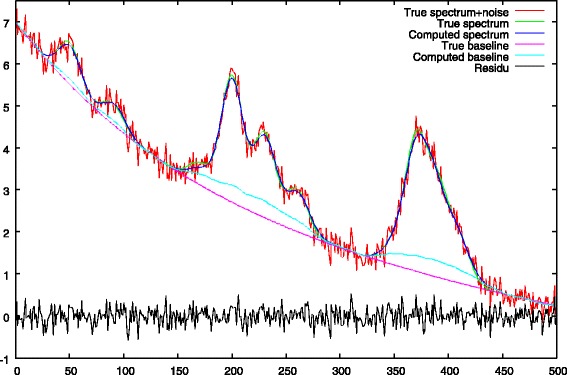
Step 1: first baseline approximation with *λ*_1_>0, comparison between the true baseline. We notice an ascent of the baseline under the peaks

As explained in “Final model formulation” section, page 8, the joint baseline computation and peak deconvolution is performed in two steps:

1. a first resolution of Eq. 20 is performed with a strong *λ*_1_ penalty. The role of this step, is to obtain peak centers from the noisy spectrum. This set of peak centers is denoted by *Ω*. This step is illustrated in Figs. 4 and 5.

**Fig. 5:**
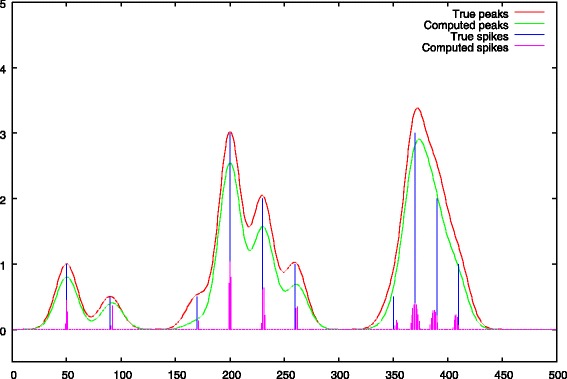
Step 1: peak selection, comparison between the true peak support and the deconvolved peaks. The negative impact of a strong *λ*_1_ penalty is showed, the peak heights are underestimated
2. a second resolution, is then performed with a null *λ*_1_ penalty. This penalty is replaced by the restricted support *Ω* for peak centers we found in the first step. The role of this step is to correct peak heights and baseline values which were biased by the presence of the strong *λ*_1_ penalty of the first step. Illustrations are given in Figs. 6 and 7.

**Fig. 6:**
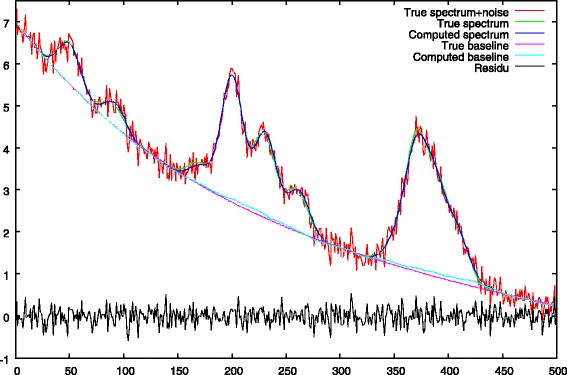
Step 2: final baseline approximation with *λ*_1_=0 and *Ω* peak support regularization. The baseline under the peaks has been corrected

**Fig. 7:**
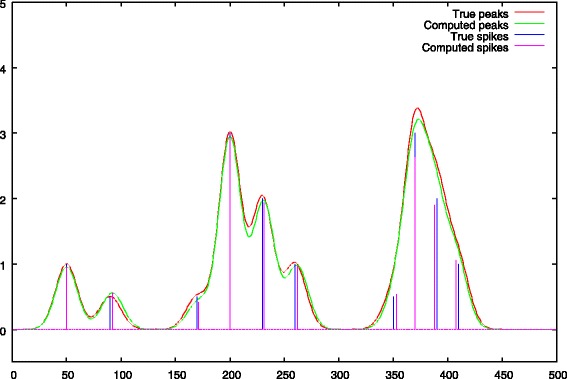
Step 2: peak debiasing, comparison between the true peak support and the deconvolved peaks

The baseline approximation obtained at the end of the first step is given in Fig. 4. This figure shows a common flaw of most of the baseline removal methods which is an ascent of the baseline under the peaks.

The deconvolved peaks obtained after the first step are given in Fig. 5. We notice the negative impact of a strong *λ*_1_ penalty which leads to an underestimation of the peak heights. The figure also shows that the deconvolved solution is less spiky in regions of strong peak overlaps (right part of the spectrum).

The baseline computed after the second step is shown in Fig. 6. During this second stage, the *λ*_1_ penalty is removed and replaced by a restricted support *Ω* computed using Eq. 16. We see that the ascent of the baseline under the peaks has been corrected (c.g. x-axis ranges from 150 to 250 and from 350 to 400).

The new peak heights are shown in Fig. 7. The main role of this second stage is to debias peak heights. Compared to Fig. 5 we can see that this objective is quite well fulfilled.

### Comparison with the usual sequential approach

In this second part we provide a comparison between our new method and a common procedure used to extract peaks. The objective is to validate the approach and to see if there are some advantages to use the joint baseline computation and peak deconvolution over more traditional approaches. Our method essentially depends on 3 hyper-parameters *λ*_1_,*λ*_2_ and *μ*. The parameter *λ*_2_ is of minor importance and is constantly set to 0.1. The two other hyper-parameters are:

1. the *λ*_1_ parameter enforcing the sparsity of the solution **x**_*p*_,
2. the *μ* parameter enforcing the smoothness of the baseline **x**_*b*_.

In order to perform a fair comparison with another approach we tried to stick to an approach that also only uses two hyper-parameters. The usual approaches in spectra processing generally chain at least two procedures [4]. In peculiar, the baseline subtraction procedure is followed by a peak picking procedure.

1. Baseline subtraction: the Statistics-sensitive Non-linear Iterative Peak-clipping (SNIP) algorithm [40–42] is an efficient algorithm to compute spectrum baseline. It generally gives good results and is easy to implement. It uses only one parameter, the window width *m*_SNIP_, but requires a smoothed spectrum. To smooth the spectrum we use a Savitzky-Golay filter [6, 7]. The idea is to locally fit the spectrum by a polynomial. This least-squares fitted polynomial of degree *d* is computed using *m*_SG_ points of a running window. For each window position, the spectrum value at the window center is then replaced by the polynomial value. In order to have only one parameter, a fixed window width *m*_SG_=39 is used for the smoothing step.
2. Peak picking: once the baseline has been subtracted from the original spectrum, there are a large panel of methods to extract peaks [4]. We can mention simple thresholding [42], second derivative computation using Savitzky-Golay filters, extraction from wavelet coefficients or deconvolution. For our comparison we are more interested by deconvolution-like methods [21, 22] which are closer to our approach. By consequence we have decided to extract peaks thanks to the usual sparse deconvolution problem:
  26 $$ \arg\min\limits_{\mathbf{x}_{p}\ge0} \frac{1}{2}\big\|\mathbf{y} - \mathbf{L}\mathbf{x}_{p}\big\|^{2}_{2} + \lambda_{1} \big\|\mathbf{x}_{p}\|_{1} + \frac{\lambda_{2}}{2} \big\|\mathbf{x}_{p}\big\|_{2}^{2}  $$
  As for our method, the coefficient *λ*_2_ is not critical and we set it to a constant value *λ*_2_=0.1. The remaining *λ*_1_ is our second free parameter. To solve Eq. 26 we use the same Barzilai-Borwein solver, detailed in Algorithm 2 and the same two-steps procedure which consists in peak selection (high *λ*_1_) and peak heights debiasing (*λ*_1_=0).

To compare the two approaches we perform a systematic grid search for the two hyper-parameters. The grid for our new method is given Table 2, whereas Table 3 is used by the usual method, that sequentially removes the baseline and then performs the peak deconvolution.

**Table 2:** Our method: used grid for parameter search

Parameters	Min	Max	Step
*μ*	100	4000	100
*λ*_1_	0	4	0.2

*λ*_2_=0.1 is constant

**Table 3:** Usual method, baseline removal then peak deconvolution: used grid for parameter search

Parameters	min	max	step
*m*_SNIP_	20	40	2
*λ*_1_	1	15	0.2

*λ*_2_=0.1 and *m*_SG_=39 are constant

For each processed spectrum we perform a systematic parameter grid search and report the best result obtained by the two methods. To find the best solution we compare the deconvolved peaks with the ground truth of our synthetic data. We use the following procedure to compare these two peak lists. As defined in our model, a peak list is stored in a sparse vector **x**_*p*_. To each nonzero component $\mathbf {x}_{p}\left [i\right ]$ corresponds a peak at position *i* and height $x_{p}\left [i\right ]$. Like computed peak centers and ground truth peak centers might not be perfectly aligned we reconstruct a continuous signal by convolving the two peak lists by the known peak shape *p*. This blurring is required later to compute the Euclidean distance between these two peak lists. This reconstruction is performed by the following matrix-vector products: $L\mathbf {x}_{p}^{\star }$ for the ground truth peak list and $L\hat {\mathbf {x}}_{p}$ for the peaks extracted by the algorithm. Using these vectors we can now compute a normalized Euclidean distance defined by:

$$\mathcal{E}_{e} =\frac{\big\|\mathbf{L}\left(\widehat{\mathbf{x}}_{p}-\mathbf{x}_{p}^{*}\right)\big\|_{2}}{\big\|\mathbf{L}\mathbf{x}_{p}^{*}\big\|_{2}} $$

**Fig. 8:**
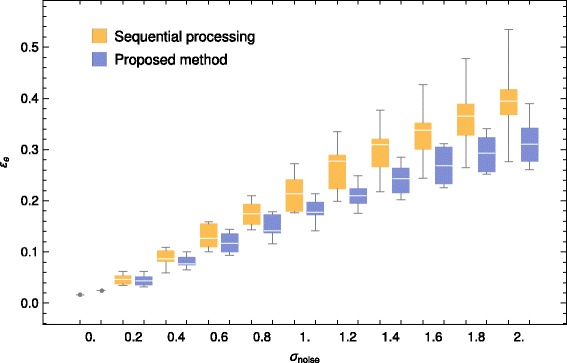
Box plot of the results presented in Table 4. $\mathcal {E}_{e}$ merit factor, lower is better

For each noise level and each algorithm the computations are performed 10 times, each time with a different noise realization. From these 10 replica we report the obtained mean and standard deviation of the $\mathcal {E}_{e}$ merit factor. The results are given in Table 4 and plotted in Fig. 8.

**Table 4:** Comparison between the two methods, for convex baseline *s*=−1

*σ*_*noise*_	Sequential processing	Proposed method
	$\mathcal {E}_{e}\times 10^{-3}$	$\mathcal {E}_{e}\times 10^{-3}$
0.	*15.*	27.6
0.2	47.8±8.76	*45.2* ±*9.12*
0.4	88.6±15.6	*81.6* ±*11.9*
0.6	131.±21.4	*118.* ±*18.*
0.8	176.±26.	*154.* ±*20.1*
1.	217.±33.7	*184.* ±*21.3*
1.2	262.±45.7	*215.* ±*25.5*
1.4	295.±48.2	*245.* ±*29.4*
1.6	330.±52.5	*275.* ±*32.7*
1.8	360.±59.8	*300.* ±*38.2*
2.	390.±70.4	*322.* ±*44.3*

$\mathcal {E}_{e}$ merit factor, lower is better. Apart from the noise free signal, the joint approach outperforms the sequential one for all noise levels

The italic means the best result (when comparing the two methods)

On this example the joint evaluation of the baseline and of the deconvolved peaks outperforms the sequential approach in nearly all configurations except for the noise-free case. In this condition, as we are using synthetic data, a perfect reconstruction of the “ground truth” spectrum is possible. By consequence, the reconstruction error is very small. To explain this result, we think that our iterative solver had stopped its iterative resolution too early.

**Fig. 9:**
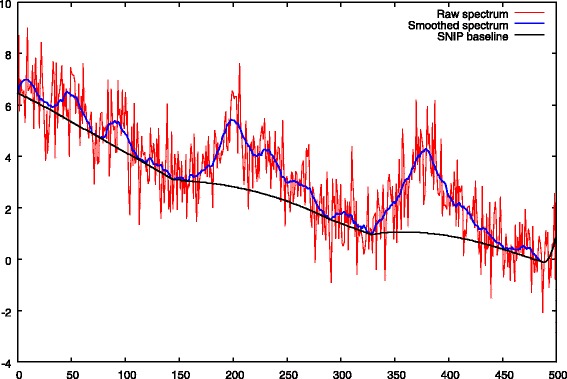
Details of the Savitzky-Golay smoothing + SNIP baseline, *σ*_*noise*_=1,*m*_SNIP_=28. The baseline removal can introduce serious artifacts. In a sequential approach these artifacts directly affect the peak picking algorithm

To get more intuition on what is happening we provide Fig. 9 illustrating the baseline computation using the SNIP algorithm.

As explained the SNIP algorithm needs to work on a previously smoothed spectrum. This smoothing is performed using a Savitzky-Golay filter. This sequential approach, smoothing then SNIP baseline, can introduce an important bias on the computed baseline. This error is then transferred to the peak picking algorithm. The result can be a poor peak extraction.

**Fig. 10:**
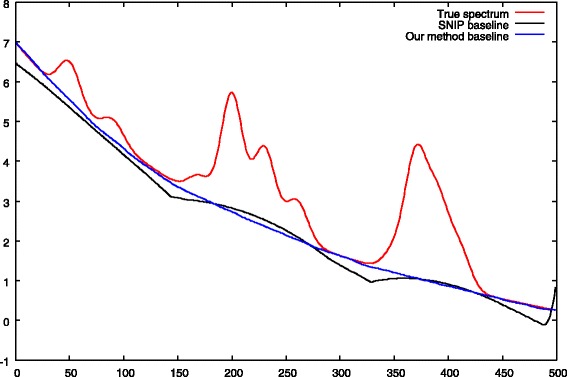
Computed baselines comparison. The blue curve is the baseline obtained by our method whereas the black one is the baseline computed by the SNIP algorithm. Our method is able the recover a nearly exact baseline unlike the usual one that presents artifacts and ascent below peaks. This result is for *σ*_*noise*_=1, *s*=−1 (convex baseline) and parameters given in Table 4

Baselines obtained by our method and by the classical SNIP algorithm are shown in Fig. 10.

We see that the simultaneous baseline and deconvolution approach allows a nearly perfect reconstruction of the baseline. Unlike our approach, the SNIP algorithm suffers from initial filtering of the spectrum and presents baseline ascent below peaks.

### Real data

**Fig. 11:**
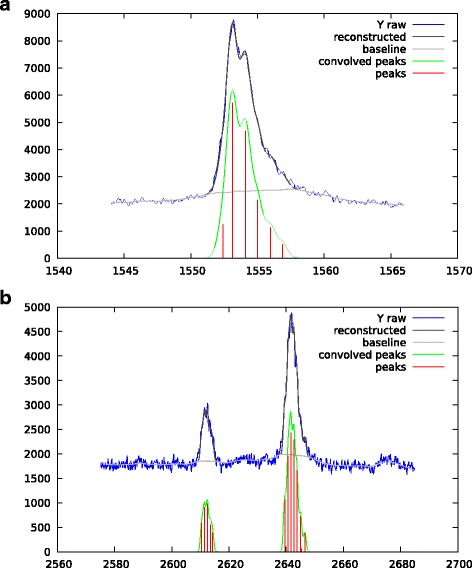
Low resolution MALDI-ToF spectrum, isotopic motifs deconvolution obtained for tight Gaussian peak shape of *σ*_peak_=0.4 *m*/*z*

We present some results obtained by our algorithm on real MALDI-ToF mass spectra. The Fig. 11 shows the peaks obtained when the low resolution spectrum is deconvolved with a tight peak shape. We have used a Gaussian peak shape with *σ*_peak_=0.4 *m*/*z*. The goal is to evaluate the behavior of the algorithm when we try to deconvolve isotopic motifs. The notable result here is to see that the returned peak centers are approximately spaced by 1 *m*/*z* which is the expected value. This result is encouraging as this information was not provided to the algorithm to perform its deconvolution.

**Fig. 12:**
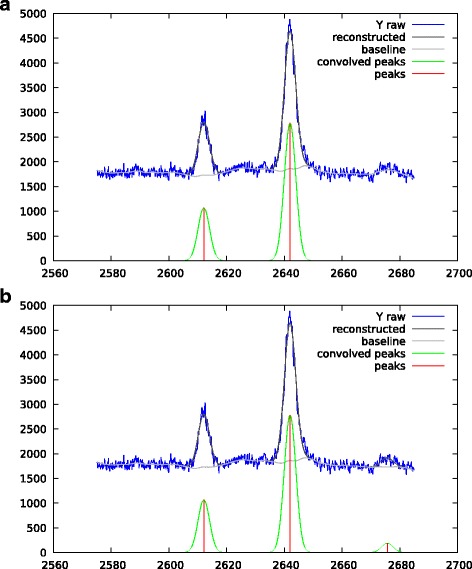
Low resolution MALDI-ToF spectrum, deconvolution obtained when considering unresolved isotopic motifs as an unique peak. The Gaussian peak shape is *σ*_peak_=2 *m*/*z*. The regularization is *λ*_1_=1 for the top plot and *λ*_1_=0.5 for the bottom one

However isotopic motif deconvolution without using any extra information (like an expected 1 *m*/*z* spacing between peaks) can lack robustness. That is the reason why it is certainly safer to use a wider peak shape to model the unresolved isotopic motif as a whole. This is illustrated by Fig. 12. We have run the algorithm twice. The first run used a sparsity promoting regularization of *λ*_1_=1, the second run used *λ*_1_=0.5.

**Fig. 13:**
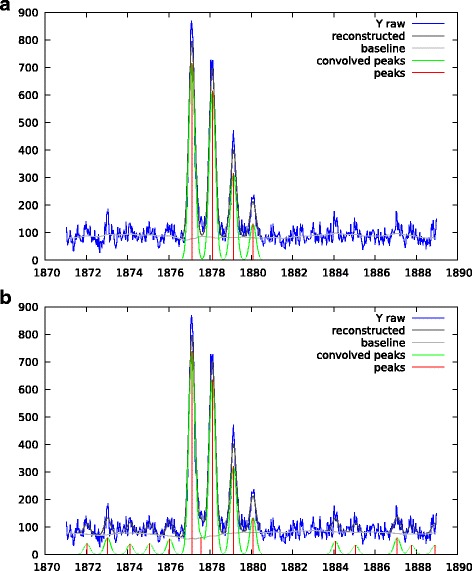
High resolution MALDI-ToF spectrum, peak extraction with a Gaussian peak shape of *σ*_peak_=0.15 *m*/*z*. The regularization is *λ*_1_=0.5 for the top plot and *λ*_1_=0.2 for the bottom one

The developed algorithm can also be used with high resolution MALDI-ToF mass spectrum (reflectron mode). Figure 13 gives such an example. A Gaussian peak shape with *σ*_peak_=0.15 *m*/*z* is used for the deconvolution. As before, we have used two values for the sparsity promoting regularization. The first run used *λ*_1_=0.5, the second run used *λ*_1_=0.2. We clearly see the impact on the results, in the first case only the main peaks are extracted, in the second one very small peaks are also extracted. For the moment this *λ*_1_ parameter has to be manually tuned by the user.

It is generally quite difficult to evaluate peak picking methods on real spectra because we do not have the ground truth at our disposal. However, in our case we have expensively used the proposed approach in the BHI-PRO project. The study [39] compares our method against a more usual one on mass spectra of spiked proteins. This study quantifies the algorithmic part of the variance of the measured protein abundances and shows a clear gain in favor of our algorithm.

## Conclusions

We have introduced a new method for peak deconvolution. This new approach jointly performs baseline computation and peak deconvolution. The baseline equation can be solved in a closed form and is substituted into the deconvolution equation. The new deconvolution equation contains a linear operator **A**_*μ*_ acting as a smoothed second order derivator. The problem of boundaries is exposed and tackled rigorously leading to a modified equation. The problem is efficiently solved by the projected Barzilai-Borwein algorithm. A comparison with a traditional approach relying on a sequential baseline removal and peak picking is detailed. The benefits of our new approach are put in evidence, including a better baseline approximation avoiding ascent below the large peaks, and a better reconstruction of the deconvolved peaks. Finally the method has been tested on real data.

## Perspectives

There are two main directions we want to explore. The first direction is to go one step further in a joint processing approach by allowing automatic adjustment of the peak shape function. Ideally, with such an approach, the baseline, the peak shape and the deconvolved peaks would be computed jointly. The second direction would be to devise a procedure for automatic tuning of the two main hyper-parameters. The resulting algorithm would be of great value to process a large number of spectra in batch mode.

## Appendix

### Smoothness and convolution matrix

To enforce baseline **x**_*b*_ smoothness the first order finite differences matrix **D** of dimension (*n*−1)×*n*

$$\mathbf{D} = \left(\begin{array}{rrrrrr} 1 & -1 & & & & \\ & 1 & -1 & & & \\ & & \ddots & \ddots & & \\ & & & 1 & -1 & \\ & & & & 1 & -1 \end{array} \right) $$

has been introduced. For ease of reading we also provide the **D**^*t*^**D** matrix expression. Its dimension is *n*×*n* and its components are:

$$\mathbf{D}^{t}\mathbf{D} = \left(\begin{array}{rrrrr} 1 & -1 & & & \\ -1 & 2 & -1 & & \\ & \ddots & \ddots & \ddots & \\ & & -1 & 2 & -1 \\ & & & -1 & 1 \end{array} \right) $$

The matrices **D**^*t*^**D** and $\mathbf {B}_{\mu }=\mathbf {I}_{b}+\mu \mathbf {D}^{t}\mathbf {D}$ are tridiagonal matrices. Hence the associated matrix-vector products can be computed efficiently. The Thomas algorithm [43], which is a Gaussian elimination optimized for tridiagonal matrices, also provides an efficient way to compute the matrix-vector products associated to the $\mathbf {A}_{\mu }= \mathbf {I}_{b}-\mathbf {B}_{\mu }^{-1}$ operator.

In the presentation the convolution product between the peak list and peak shape function, **x**_*p*_∗*p*, is often represented by the **Lx**_*p*_ matrix-vector product. However, in practical computations the **L** matrix is never explicitly formed and the convolution product is computed directly using a specialized subroutine.

### Derivation of reduced objective

This appendix focuses on the reduced criterion Eq. 10.

Let *φ* be a function which takes as input a vector **z** of size *n* and produces as output the scalar quantity

$$\varphi(\mathbf{z}) = \frac{1}{2} \mathbf{z}^{t} \mathbf{Q} \mathbf{z} + \mathbf{q}^{t} \mathbf{z} + q_{0} $$

where *q*_0_ is a scalar, **q** is a vector of size *n* and **Q** is a strictly positive-definite symmetric matrix of size *n*×*n*. The minimizer of *φ* is $\hat {\mathbf {z}} = -\mathbf {Q}^{-1}\mathbf {q}$ and the minimum is

$$\varphi\left(\hat{\mathbf{z}}\right) = -\frac{1}{2}\mathbf{q}^{t}\mathbf{Q}^{-1}\mathbf{q} + q_{0} $$

If we introduce the gradient ∇*φ*(**z**)=**Qz**+**q** and the Hessian ∇^2^*φ*(**z**)=**Q** we notice that the previous relation takes the following form:

27 $$ \varphi(\hat{\mathbf{z}}) = -\frac{1}{2}\nabla \varphi(\mathbf{0})^{t} \left[\nabla^{2}\varphi(\mathbf{0})\right]^{-1}\nabla\varphi(\mathbf{0}) + \varphi(\mathbf{0})  $$

If we consider Eq. 6 as a function of **x**_*b*_

$$\mathbf{x}_{b}\rightarrow \mathcal{J}\left(\mathbf{x}_{b},\mathbf{x}_{p}\right) = \frac{1}{2} \big\|\bar{\mathbf{y}} - \mathbf{x}_{b} \big\|^{2}_{2} + \frac{\mu}{2} \big\|\mathbf{D}\mathbf{x}_{b}\big\|^{2}_{2} + q_{0} $$

where

$$\begin{array}{*{20}l} \bar{\mathbf{y}} &\, =\,\, \mathbf{y} - \mathbf{L} \mathbf{x}_{p} \\ q_{0} &\, =\,\, \lambda_{1} \big\|\mathbf{x}_{p}\big\|_{1} + \frac{\lambda_{2}}{2} \big\|\mathbf{x}_{p}\big\|_{2}^{2} \end{array} $$

and

$$\begin{array}{*{20}l} \mathcal{J}(\mathbf{0},\mathbf{x}_{p}) &\, = \,\, \frac{1}{2}\|\bar{\mathbf{y}}\|{~}^{2}_{2}+q_{0} \\ \nabla_{\mathbf{x}_{b}}\mathcal{J}(\mathbf{0},\mathbf{x}_{p}) &\, =\,\, -\bar{\mathbf{y}} \\ \nabla^{2}_{\mathbf{x}_{b}}\mathcal{J}(\mathbf{0},\mathbf{x}_{p}) &\, =\,\, \mathbf{B}_{\mu} \end{array} $$

then application of Eq. 27 directly leads to Eq. 10:

$$\widetilde{\mathcal{J}}\left(\mathbf{x}_{p}\right) = \mathcal{J}\left(\hat{\mathbf{x}}_{b},\mathbf{x}_{p}\right) + \mathbf{C} $$

with a constant term $\mathbf {C}=-\frac {1}{2}\mathbf {y}^{t} \mathbf {A}_{\mu } \mathbf {y}$.

### Expression of $\underset {\mu \rightarrow \infty }{\lim } \mathbf {A}_{\mu }$

The matrix **D**^*t*^**D** is real and symmetric, hence it admits an eigen decomposition

$$\mathbf{D}^{t}\mathbf{D} = \mathbf{V}\mathbf{\Delta}\mathbf{V}^{*}=\sum\limits_{k=1}^{n} \nu_{k} \mathbf{v}_{k} \otimes \mathbf{v}_{k} $$

where **Δ** is a diagonal matrix of eigenvalues *ν*_*k*_ and **V** is an orthonormal matrix. Each column *k* of *V* is an eigenvector **v**_*k*_.

Let’s write down two basic statements about eigenpair $\left (\nu,\mathbf {v}\right)$ of any square matrix *M*:

- the first one, where *I* denotes the identity matrix, is:

$$M\mathbf{v}=\nu\mathbf{v}\Leftrightarrow (I+M)\mathbf{v}=(1+\nu)\mathbf{v} $$

- the second one, that requires *M* to be invertible (hence *ν*≠0), is:

$$M\mathbf{v}=\nu\mathbf{v}\Leftrightarrow M^{-1}\mathbf{v}=\frac{1}{\nu}\mathbf{v} $$

Now observe that like $\mathbf {I}_{b}+\mu \mathbf {D}^{t}\mathbf {D}$ is a strictly diagonally dominant matrix it is invertible [44]. Using the two previously enumerated relations it is easy to show that this matrix admits the following eigen decomposition:

$$\left(\mathbf{I}_{b}+\mu \mathbf{D}^{t}\mathbf{D}\right)^{-1} = \sum\limits_{k=1}^{n} \frac{1}{1+\mu\nu_{k}} \mathbf{v}_{k} \otimes \mathbf{v}_{k} $$

By construction (first order finite differences) the product of the matrix **D** with a constant vector is the null vector. It follows that we have $\mathbf {D}^{t}\mathbf {D}\,\mathbf {v}_{1}=\mathbf {0}$ where $\mathbf {v}_{1}=\frac {1}{\sqrt {n}}\mathbb {1}_{n}$. Thus **v**_1_ is the normalized eigen vector associated to the *ν*_1_=0 eigenvalue. Moreover, as **D**^*t*^**D** is an *unreduced*^2^ real symmetric tridiagonal matrix, all its eigenvalues are simples ([45], Lemma 7.7.1). By consequence we can write:

$$\left(\mathbf{I}_{b}+\mu \mathbf{D}^{t}\mathbf{D}\right)^{-1} = \mathbf{v}_{1} \otimes \mathbf{v}_{1} + \sum\limits_{k=2}^{n} \frac{1}{1+\mu\nu_{k}} \mathbf{v}_{k} \otimes \mathbf{v}_{k} $$

with *ν*_*k*_≠0 for all *k*≥2. It is then trivial to compute the limit:

$$\lim\limits_{\mu\rightarrow\infty} \left(\mathbf{I}_{b}+\mu \mathbf{D}^{t}\mathbf{D}\right)^{-1} = \mathbf{v}_{1} \otimes \mathbf{v}_{1} = \frac{1}{n}\mathbb{1}_{n\times n} $$

which is a square matrix of dimensions *n*×*n* with all entries equal to 1.

From the definition of **A**_*μ*_ in Eq. 11 we get the expected result:

$$\lim\limits_{\mu\rightarrow\infty} [ \mathbf{A}_{\mu}]_{i,j}=\delta_{i,j}-\frac{1}{n} $$

with *δ*_*i*,*j*_ equals to 1 if *i*=*j* and 0 otherwise.

### Boundary correction

Our boundary correction approach exploits the opportunity to convert an equality constrained problem

28 $$ \arg\min\limits_{\mathbf{x},\ \mathbf{x}[k]=\bar{y}_{k}} \frac{1}{2}\mathbf{x}^{t}\mathbf{Q}\mathbf{x}+ \mathbf{x}^{t} \mathbf{q}  $$

into a unconstrained modified problem

29 $$ \arg\min\limits_{\mathbf{x}}\frac{1}{2} \mathbf{x}^{t} \widetilde{\mathbf{Q}} \mathbf{x} + \mathbf{x}^{t} \widetilde{\mathbf{q}}  $$

Let begin by writing the Lagrangian [38] associated to Eq. 28.

$$\mathcal{L}(\mathbf{x},\lambda_{k})=\frac{1}{2} \mathbf{x}^{t} \widetilde{\mathbf{Q}} \mathbf{x} + \mathbf{x}^{t} \widetilde{\mathbf{q}}+\lambda_{k}\mathbf{e}_{k}^{t}\left(\mathbf{x}\left[k\right]-\bar{y}_{k}\right) $$

The KKT conditions are then easily obtained by differentiating the Lagrangian and by imposing constraint satisfaction:

$$\left(\nabla_{\mathbf{x}} \mathcal{L}\left(\mathbf{x},\lambda_{k}\right)=\mathbf{Q}\mathbf{x}+\mathbf{q}+\lambda_{k}\mathbf{e}_{k}=0\right)\wedge\left(\mathbf{x}\left[k\right]=\bar{y}_{k}\right) $$

where *λ*_*k*_ is the Lagrangian multiplier associated to the constraint $\mathbf {x}[k]=\bar {y}_{k}$ and **e**_*k*_ the vector of component *δ*_*k*_(*i*). We can expand the $\nabla _{\mathbf {x}} \mathcal {L}(\mathbf {x},\lambda _{k})=0$ equation to get:  It is then obvious that if we take:

$$\lambda_{k}=-\left(\mathbf{v}^{t}\mathbf{x}^{-} + \bar{y}_{k}\mathbf{Q}(k,k) + \mathbf{w}^{t}\mathbf{x}^{+} + \mathbf{q}[\!k]\right) $$

then the constraint $\mathbf {x}[k]=\bar {y}_{k}$ is fulfilled. Substituting this into the $\nabla _{\mathbf {x}} \mathcal {L}(\mathbf {x},\lambda _{k})=0$ equation we get:  This equation is usable but its drawback is that we have lost the symmetry of the modified **Q** matrix. This can be fixed by another modification of the vector term **q**. Exploiting the fact that by construction $\mathbf {x}[\!k]=\bar {y}_{k}$ we have the following equivalent system: 
30

By identification we can write down the announced result:

$$\arg\min\limits_{\mathbf{x}}\frac{1}{2}\mathbf{x}^{t} \widetilde{\mathbf{Q}} \mathbf{x} + \mathbf{x}^{t}\widetilde{\mathbf{q}} $$

which is an unconstrained system for which the minimizer satisfies $\mathbf {x}\left [k\right ]=\bar {y}_{k}$. For presentation clarity we only have considered one constraint, but this scheme can be used sequentially for any set of constraints

$$\left\{\mathbf{x}\left[k\right]=\bar{y}_{k},\ k\in K\right\} $$

Now we can see how to use this technique to our deconvolution problem. We must go back to Eq. 8:

$$\nabla_{\mathbf{x}_{b}} \tilde{J} = \underbrace{(\mathbf{I}_{b}+ \mu \mathbf{D}^{t}\mathbf{D})}_{\mathbf{B}_{\mu}} \mathbf{x}_{b} + \mathbf{L}\mathbf{x}_{p} -\mathbf{y} $$

By identification the role of the matrix **Q** is played by the **B**_*μ*_ matrix. Furthermore all the modifications done on the vector **q** can be transposed to modifications associated to the −**y** vector.

As an illustrative example, we consider the most usual case that consists of imposing the left $\bar {y}_{1}$ and right $\bar {y}_{n}$ baseline values at boundaries. Direct application of Eq. 30 provides the result. The modified **B**_*μ*_ matrix is:

31 $$ {}{\widetilde{\mathbf{B}}_{\mu}\,=\, \left(\! \begin{array}{cccccccc} 1+\mu & 0 & & & & & & \\ 0 & 1+2\mu & -\mu & & & & & \\ & -\mu & 1+2\mu & -\mu & & & & \\ & & \ddots & \ddots & \ddots & & & \\ & & & -\mu & 1+2\mu & -\mu & & \\ & & & & -\mu & 1+2\mu & 0 & \\ & & & & & 0 & 1+\mu \end{array} \!\!\!\right)}  $$

The modified **y** vector is:

32 $$ \widetilde{\mathbf{y}}= \left(\begin{array}{c} (1+\mu) \bar{y}_{1} \\ \mathbf{y}\left[2\right]+\mu \bar{y}_{1} \\ \mathbf{y}\left[3\right] \\ \vdots \\ \mathbf{y}\left[n-2\right] \\ \mathbf{y}\left[n-1\right]+\mu \bar{y}_{n} \\ (1+\mu) \bar{y}_{n} \end{array} \right)  $$

With these modifications, the modified Eq. 9 that allows to retrieve the baseline **x**_*b*_ from the deconvolved peaks **x**_*p*_ is now:

$$\mathbf{x}_{b} = \widetilde{\mathbf{B}}_{\mu}^{-1}\left(\widetilde{\mathbf{y}}-\mathbf{L}\mathbf{x}_{p}\right) $$

The modified **A**_*μ*_ operator Eq. 11 is computed as before

33 $$ \widetilde{\mathbf{A}}_{\mu}=\mathbf{I}_{b}-\widetilde{\mathbf{B}}_{\mu}^{-1}  $$

The final quadratic form Eq. 13 must be replaced by:

34 $$\begin{array}{*{20}l} \widetilde{\mathcal{J}}_{2}(\mathbf{x}_{p})\,\, = &\,\, \frac{1}{2} \mathbf{x}_{p}^{t}\left(\lambda_{2} \mathbf{I}_{p}+ \mathbf{L}^{t}\widetilde{\mathbf{A}}_{\mu} \mathbf{L}\right) \mathbf{x}_{p}+  \\ &\,\,\mathbf{x}_{p}^{t}\left(\lambda_{1} \mathbf{\mathbb{1}}_{p} - \mathbf{L}^{t}\widetilde{\mathbf{A}}_{\mu}\widetilde{\mathbf{y}} - \mathbf{L}^{t} \left(\mathbf{y}-\widetilde{\mathbf{y}}\right)\right) \end{array} $$

## Abbreviations

BB: Barzilai-Borwein
BHI-PRO: Bayesian hierarchical inversion for mass spectrometry. Application to discovery and validation of new PROtein biomarkers
CWT: Continuous wavelet transform
DWT: Discrete wavelet transform
KKT: Karush-Kuhn-Tucker
LC: Liquid chromatography
MALDI-ToF: Matrix-assisted laser desorption/ionization-time of flight
MS: Mass spectrometry
SG: Savitzky-golay
SNIP: Statistics-sensitive non-linear iterative peak-clipping
UDWT: Undecimated wavelet transform

## Footnotes

1
https://github.com/vincent-picaud/Joint_Baseline_PeakDeconv

2 No off-diagonal element is null
